# Utility of Computational Modeling in Reassessing the Threshold for Intervention and Progression into Type A Aortic Dissection

**DOI:** 10.3390/biomedicines14030696

**Published:** 2026-03-17

**Authors:** Mohammad Al-Rawi, Eric T. A. Lim, Manar Khashram, William J. Yoon

**Affiliations:** 1 CSU Engineering, Charles Sturt University, Bathurst, NSW 2795, Australia; 2 Department of Vascular & Endovascular Surgery, Waikato Hospital, Hamilton 3204, New Zealand; eric_lta@hotmail.com (E.T.A.L.); manar.khashram@gmail.com (M.K.); 3 Department of Surgery, University of Auckland, Auckland 1023, New Zealand; 4 Department of Vascular Surgery, MedStar Washington Hospital Center–Georgetown University Hospital, Washington, DC 20009, USA; william.yoon@uu.se; 5 Department of Surgical Sciences: Vascular Surgery, Uppsala University, 75185 Uppsala, Sweden

**Keywords:** CFD modeling, wall shear stress (WSS), pressure gradient, pre-aortic dissection and post-aortic dissection type A

## Abstract

**Background:** Assessing aortic dissection (AD) in its early stages is crucial for cardiovascular surgeons to improve patient outcomes and avoid complications associated with surgical intervention for type A aortic dissection. Initial evaluations rely on patient referrals for computed tomography (CT) scans, which involve measuring the maximum aortic diameter. **Objective:** This study aimed to improve current diagnostic thresholds for type A aortic dissection by using computational fluid dynamics (CFD) modeling to correlate hemodynamic factors related to the wall shear stress with maximum aortic diameter growth rate, offering insights into predicting AD progression and reassessing current diameter-based diagnostic criteria. **Methods:** The pre- and post-AD scan data, with an average duration of three and a half years for the 15 patients, were converted into 3D geometries. These geometries were analyzed using the transitional-turbulent CFD model. Wall shear stress (WSS), its derivatives, and the pressure gradient from the pre-AD CT scans were compared across 15 patients, grouped according to the aortic diameter growth per year. **Results:** For patients in group 1 (nine patients with normal diagnosis), pre-AD time-average wall shear stress (TAWSS) was mostly 2–4 Pa, above physiologic levels. Post-AD, values dropped below 1.5 Pa (stagnant, thrombus-prone), with oscillatory shear index (OSI) elevated (0.24–0.32). In group 2 (*n* = 6, abnormal diagnosis), post-AD TAWSS was <3 Pa (thrombosis risk), with OSI 0.1–0.31 near tear sites. These findings confirm a dual-risk profile: low TAWSS promotes thrombosis, while high TAWSS drives dissection progression. **Conclusions:** WSS parameters, such as TAWSS and OSI, can be utilized to assess the development of a dilated ascending aorta, especially for extreme maximum aortic diameter. Pre-AD analysis for some patients revealed a strong negative correlation, indicating that high shear stress in the true lumen (TL) results in a drop in diastolic pressure post-AD at the upward-going section of the aorta.

## 1. Introduction

Aortic dissection (AD) is defined as a tear involving the innermost layer, tunica intima, of the aortic wall that allows blood to flow into and separate the middle (tunica media) and outer layers (tunica externa). Based on the tear location, AD is classified as type A or B. A type A diagnosis shows the changes to the ascending aorta wall and indicates that the tear could reach the aortic arch. However, the tear location for type B occurs at the descending aorta (descending aortic dissections (DADs)) and distal to the origin of the left subclavian artery. The most common risk factors for AD include hypertension, smoking, the presence of aortic dilation/aneurysms, and genetic predispositions to aortic disease [1]. Diagnosing AD can be extremely challenging, with its common clinical symptoms often mimicking other clinical pathologies. Patients often describe the associated chest pain as one of the most severe they have ever experienced, typically of a “tearing” or “ripping” nature [2].

AD poses a high risk of mortality and morbidity to patients of different ages. Traditionally, ascending aortic dissections require emergent surgical intervention, while DADs are managed medically [2,3]. Early diagnosis of AD is crucial, as it can prevent the need for complex surgical interventions or even death [3,4]. The current incidence of AD is approximately 3.13 per 100,000 person-years [3,5]. Current guidelines have set a minimum surgical intervention threshold of 55 mm, as suggested by Isselbacher et al. [6].

Hemodynamics and mechanical stresses can weaken the aortic wall at the ascending aorta, aortic arch, and descending aorta, which can lead to an intimal tear, initiating an aortic dissection. This creates a separation in the aortic wall and splits the blood flow into a false lumen (FL) and a true lumen (TL). This, in turn, can alter the hemodynamics of blood flow within the aorta. AD usually presents acutely, although some cases can occur over a longer timeframe, either symptomatically or as incidental findings [7].

Recently, to better predict risk factors for AD, hemodynamic studies, like those using computational fluid dynamics (CFD), are increasingly used to understand and predict the effects of AD [8,9,10,11,12,13]. CFD modeling, based on aortic geometries constructed from CT scans, provides patient-specific analysis of blood flow in the aorta, offering detailed insights into hemodynamic parameters, such as time-averaged wall shear stress (TAWSS) and the oscillatory shear index (OSI), affected by the dissection. By measuring these parameters, clinicians can assess the impact of entry tears on the hemodynamics of aortic dissections, helping to predict the development/progression of AD [14].

Several publications have explored the utility of these hemodynamic parameters from CFD analysis in type A aortic dissection (TAAD) specifically. Firstly, analysis showed that the maximum TL area of the ascending aorta was negatively correlated with the risk of rupture and thus can be used as a reference point for assessing rupture risk in TAAD [15]. Using CFD analysis, blood pressure has been demonstrated to be high at exit tears in type A aortic dissection, which can lead to increased pressure in the FL, with chronic hypertension being shown to be a risk factor for the development of TAAD [16,17]. Wall shear stress (WSS) has been shown to correspond to the development of retrograde TAAD. High values of WSS were observed in CFD analyses performed at the point of entry tear and can be used as a predictive parameter for risk of retrograde TAAD [18]. Furthermore, it has been shown that WSS values > 10 Pa predispose the ascending aorta to potential endothelial cell damage and the subsequent development of TAAD [19] and potentially increased mortality, especially when the WSS is found to be twice the values observed in healthy patients [20]. From CFD analysis, TAWSS has also been observed to be useful in identifying patients at low risk of dissection progression when low TAWSS contours are seen in the FL [21,22]. Conversely, a risk of ongoing aortic wall damage or dissection is indicated if high TAWSS contours are observed [23].

In this study, 15 patients with type A-AD were computationally analyzed based on pre- and post-AD development CT scans (generating 30 3D aortic geometries) to assess the current relevance of surgical intervention thresholds. The investigation correlates literature-suggested parameters—TAWSS, OSI, and the maximum aortic diameter growth rate/year—to evaluate a patient’s progression into a type A aortic dissection and reassess how this can guide current surgical decision-making criteria for the minimum aortic diameter threshold for surgery. This study applies a reverse engineering approach, using the post-AD CT scan data to identify the locations of weakness in the aortic wall where the tear occurred. This study applies the same boundary conditions across all geometries to assess the vessel’s physical structure, such as the aortic curvature of each aorta.

## 2. Materials and Methods

This was a retrospective study conducted in a major tertiary vascular referral center in Waikato Hospital, New Zealand. All patients referred with a confirmed type A acute aortic syndrome on computed tomography (CT) angiography were identified from a prospectively collected local database.

All patients with acute aortic syndrome were identified based on CT scan image quality and the availability of post-type A aortic dissection imaging, under ethical approval number HDEC20/NTB/217. Patients with type A acute aortic syndrome were defined as those diagnosed with type A intramural hematoma (IMH), penetrating aortic ulcer (PAU), or aortic dissection.

Only patients with confirmed type A aortic dissection and prior CT imaging were included in the study. Patients with type A IMH or PAU were not suitable for computational fluid dynamics (CFD) analysis and were therefore excluded from the study.

All CT scan data were extracted from electronic hospital records and reviewed, including relevant radiology imaging reports. The most recent CT imaging prior to the type A aortic dissection event was utilized for comparison and labeled pre-AD. CT imaging following presentation with an acute type A aortic dissection was labeled post-AD. Measurements of the aortic diameter were performed using an outer-to-outer diameter measurement as per current guidelines (Czerny 2024 EACTS/STS Guidelines for Diagnosing and Treating Acute and Chronic Syndromes) [24]. Diameter measurement of the ascending aorta/aortic root was performed above the sinotubular junction, and measurement of the proximal descending thoracic aorta was performed at the level of the pulmonary trunk.

### 2.1. Geometry Construction

**Figure 1:**
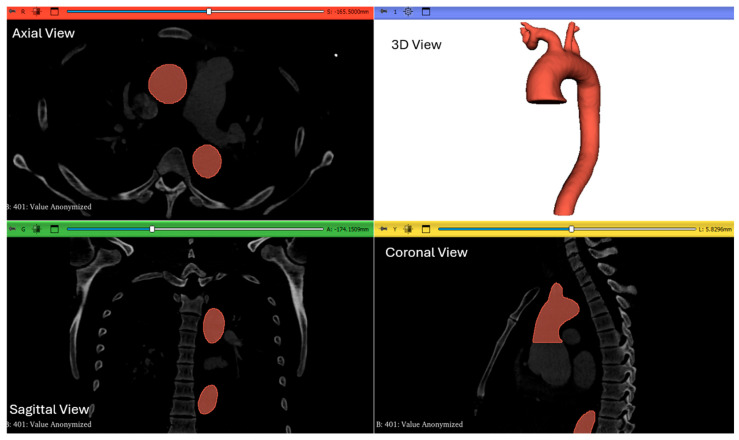
The 3D slicer (showing the axial view, sagittal view, and coronal view slices) and the 3D STL file for one of the patients pre-AD.

The CT scan images, both pre- and post-AD, were investigated and analyzed using 3D Slicer (www.slicer.org) [25]. The software was used to segment each slice and generate 3D STL files. One of the pre-AD images is illustrated in Figure 1. A manual segmentation approach was applied to isolate the aortic lumen using the Segment Editor module to ensure accurate reconstruction of branch ostia, curvature regions, and anatomically complex areas of the aorta. The segmentation was visually verified slice-by-slice in the axial, sagittal, and coronal planes. Surface smoothing was applied conservatively to remove staircase artifacts while preserving anatomical fidelity. The aorta was then generated as a three-dimensional geometry in STL format, as shown in Figure 1 (top right).

### 2.2. Computational Setup and Properties

The smoothed STL files for the 30 aortas were clipped to create flat boundary conditions at the ends of the aortic geometry and were designated as inlet and outlet boundaries. This preprocessing was performed in ANSYS SpaceClaim 2023 R2. At the inlet, a blood flow waveform was applied, while pressure waveforms were imposed at the outlets. These waveforms were embedded into Fluent as user-defined expressions developed from the literature [26].

**Figure 2:**
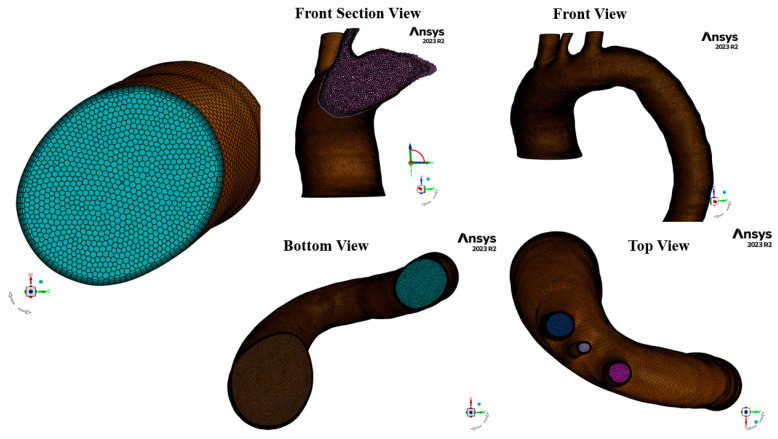
The mesh for the STL file using Fluent Meshing for one of the patients pre-AD.

Surface and volume meshing, including local refinements and boundary layer generation, was carried out in Fluent Meshing (ANSYS, Inc., Canonsburg, PA, USA) using the Watertight Geometry Workflow for all 30 aortic geometries, as shown in Figure 2. Flow analyses were performed by modeling blood as a turbulent fluid using the k-omega shear stress transport (SST) model. This model is embedded into ANSYS Fluent to activate the Reynolds-Averaged Navier–Stokes (RANS) equations. These equations are coupled with the transport equations (the turbulent kinetic energy (k)) and the specific dissipation rate (ω). Equations (1) and (2) represents the continuity equation, Equation (3) represents the momentum equation (RANS form), Equation (4) represents the turbulent kinetic energy (k) transport equation, and Equation (5) represents the specific dissipation rate (ω) transport equation.

(1) $$\frac{\partial \rho }{\partial t}=\nabla \cdot \left(\rho \vec{u}\right)=0,$$

where ρ is the blood density (kg/m^3^), $\vec{u}$ is the blood velocity vector (m/s), and $\nabla \cdot \left(\rho \vec{u}\right)$ is the divergence mass flux.

(2) $$\frac{\partial (\rho \vec{\mu })}{\partial t}+\nabla \cdot \left(\rho \vec{\mu }\vec{\mu }\right)=-\nabla p+\nabla \cdot \left[\mu \left(\nabla \vec{\mu }+\nabla {\vec{\mu }}^{T}\right)\right]-\nabla \cdot \bar{\rho \mu^{\prime }\mu^{\prime }},$$

where ∇p is the pressure gradient, and $\nabla \cdot \left[\mu \left(\nabla \vec{\mu }+\nabla {\vec{\mu }}^{T}\right)\right]$ is the viscous stress tensor, which accounts for diffusion of momentum due to blood viscosity. $-\nabla \cdot \bar{\rho \mu^{\prime }\mu^{\prime }}$ is the turbulent Reynolds stresses (from Reynolds averaging and RANS equations for the k-ω SST model, as shown in Equation (3)):

(3) $$-\rho \bar{\mu_{i}^{\prime }\mu_{j}^{\prime }}=\mu_{t}\left(\frac{\partial u_{i}}{\partial x_{j}}+\frac{\partial u_{j}}{\partial x_{i}}\right)-\frac{2}{3}\rho k\delta_{ij},$$

(4) $$\frac{\partial (\rho k)}{\partial t}+\nabla \cdot \left(\rho k\vec{u}\right)=\nabla \cdot \left[\left(\mu +\sigma_{k}\mu_{t}\right)\nabla k\right]+P_{k}-\beta *\rho k\omega ,$$

where μ is the the local Carreau viscosity in (Pa.s), $\mu_{t}$ is the turbulent (eddy) viscosity, $\sigma_{k}$ is the turbulent Prandtl number for k, and $P_{k}$ is the turbulence production term. For the SST model, β*=0.09.

(5) $$\frac{\partial (\rho \omega )}{\partial t}+\nabla \cdot \left(\rho \omega \vec{u}\right)=\nabla \cdot \left[\left(\mu +\sigma_{\omega }\mu_{t}\right)\nabla \omega \right]+\alpha \frac{\omega }{k}P_{k}-\beta \rho \omega^{2}+2(1-F_{1})\rho \sigma_{\omega 2}\frac{1}{\omega }\nabla k\cdot \nabla \omega ,$$

where $\alpha \frac{\omega }{k}P_{k}$ is the coupling between turbulence production and ω, $F_{1}$ blending function (activates near-wall corrections), and $2(1-F_{1})\rho \sigma_{\omega 2}\frac{1}{\omega }\nabla k\cdot \nabla \omega$ presents the cross-diffusion for the SST.

To provide more accurate results for assessing thrombus risk associated with aortic dissection development, blood was modeled as a non-Newtonian fluid (ρ = 1060 kg/m^3^). The viscosity was based on the Carreau model, which was activated in ANSYS Fluent and solved using Equation (6) [26,27,28,29,30].

(6) $$\mu \left(\dot{\gamma }\right)=\mu_{\infty }+\left(\mu_{o}-\mu_{\infty }\right)[1+{\left(\lambda \dot{\gamma }\right)}^{2}{]}^{\frac{n-1}{2}},$$

The literature recommended using the k-ω SST model due to its ability to capture the turbulent and non-Newtonian nature of blood flow in the presence of abnormal flow behavior in the aorta [28,29,30,31]. This model is also recommended to address the transition from laminar to turbulent flow as blood reaches the aortic arch and bifurcations.

### 2.3. Boundary Conditions

**Figure 3:**
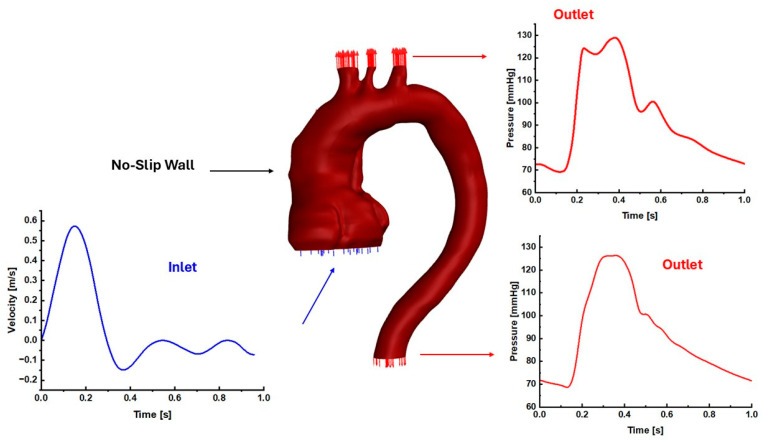
The computational domain and the boundary conditions for one patient post-AD.

The inlet and outlet boundary conditions were based on our published data for a healthy patient [31], as shown in Figure 3. The inlet boundary condition for each model was set to a velocity waveform, and the outlets were set to pressure waveforms with the assumption of a no-slip artery wall, as shown in Figure 3. Due to the unavailability of patient-specific boundary conditions and to evaluate the hemodynamic parameters while accounting for variations in aortic geometry, the literature-based blood flow and pressure waveform boundary conditions were applied to examine how they propagate through the aortic geometry. The CFD was based on using a second-order upwind scheme for spatial discretization and the SIMPLE algorithm (Semi-Implicit Method for Pressure-Linked Equations) for pressure-velocity coupling. For the run calculation, we set the number of time steps at 300 with a time step size of 0.01 s for three consecutive cardiac cycles with a 70 bpm heart rate and a maximum number of iterations of 30 [27]. The convergence criteria for the solutions were considered when the residuals for the continuity and velocity achieved $1\times 10^{-5}.$ Each of the CFD analyses for all these models (30 aorta cases) was performed under ANSYS Fluent 2023R2 using the following local computer: 13th Gen Intel(R) Core (TM) i7-13700F, 2.10 GHz, and 64 GB RAM.

### 2.4. Hemodynamic Analysis

To assess the impact of aortic dissection development, wall shear stress (WSS) data in the x, y, and z directions were exported as text files for all 30 aortic models. These data were then processed in MATLAB R2024a to calculate the time-averaged wall shear stress (TAWSS), using Equation (7), and the oscillatory shear index (OSI), using Equation (8), at a temporal resolution of 0.01 s. TAWSS and OSI provide insights into regions where the aortic wall is subjected to abnormal shear stresses caused by changes in geometry and the presence of intimal tears. Regions of low TAWSS and high OSI have been associated with adverse aortic complications and an increased risk of dissection progression.

(7) $$TAWSS=\frac{1}{T}\int_{0}^{T}\left|\tau \right|dt,$$

(8) $$OSI=0.5\left[1-\left(\frac{\left|\int_{0}^{T}\tau dt\right|}{\int_{0}^{T}\left|\tau \right|dt}\right)\right],$$

*T* was set to 3, corresponding to three cardiac cycle periods for each aorta, and τ represents the instantaneous WSS in the x, y, and z directions exported from Fluent at each time step. This process involved analyzing 300 text files per aorta to generate the contours for TAWSS (Pa) and OSI (-).

## 3. Computational Validation

### 3.1. Mesh Test Analysis and Sensitivity Test

The mesh test analysis for the 30 aortic geometries (pre- and post-AD) was performed using the Fluent Watertight Geometry meshing option and unsteady flow simulations in ANSYS Fluent. Four different mesh densities were tested, as shown in Table 1 for patient 1 in group 1. Mesh 2 (M2) produced highly acceptable results, with a total of 963,241 cells.

**Figure 4:**
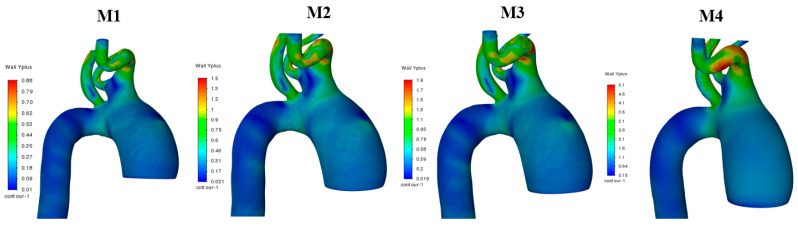
The wall $Y^{+}$ values for the four meshes of the aorta in patient 1 in group 1.

A mesh-sensitivity test was performed to evaluate grid independence by assessing whether changes in mesh density significantly affected the solution. Mesh quality and suitability for simulating near-wall turbulence were examined using the dimensionless wall $Y^{+}$ parameter, defined as

(9) $$Y^{+}=\frac{\rho \mu_{\tau }y}{\mu },$$

where $\mu_{\tau }$ is the friction velocity, and y is the distance from the wall to the center of the first computational cell. The value of $Y^{+}<1.9$, achieved in this study (Figure 4), is generally considered ideal for simulations using a turbulent model.

**Figure 5:**
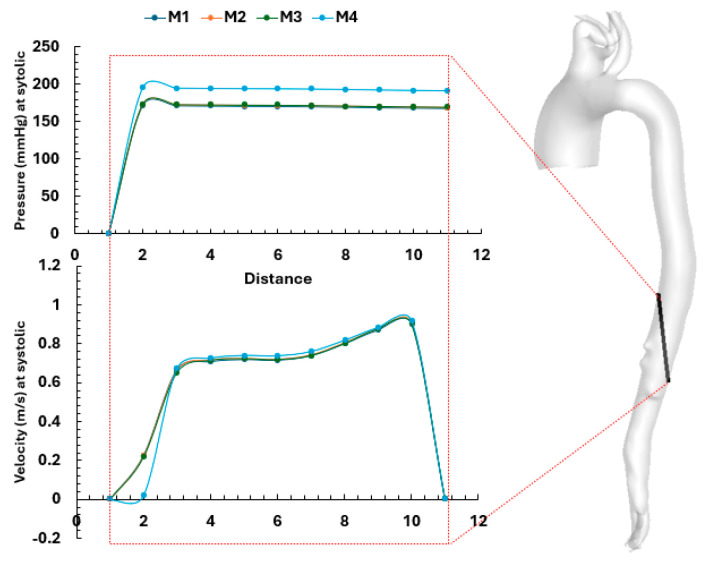
Pressure and velocity values along a line selected downstream of the descending aorta, showing the consistency of the mesh results.

In addition, for the same patient, a line was selected downstream of the descending aorta to plot the pressure and velocity values along it for the steady case analysis as shown in Figure 5. The results showed that meshes M1–M3 produced similar outcomes, whereas M4 exhibited deviations due to the lower number of cells.

### 3.2. Validation

**Figure 6:**
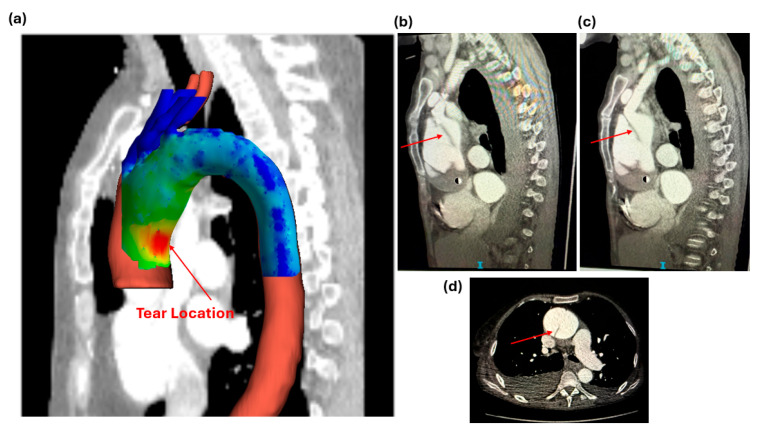
(**a**) Pre-AD diagram showing the shear stress location for the tear prediction, (**b**) sagittal view of the post-AD showing the tear location (image 1), (**c**) sagittal image at a different slice (image 2), and (**d**) axial view.

Following mesh accuracy verification for each patient-specific aortic model, parameters of interest were extracted from the ANSYS Fluent analysis of the pre-AD models for validation against the corresponding post-AD cases. For patient 1 in group 1, the shear stress contours (Figure 6) confirmed agreement with the observed tear location in the ascending aorta. This validation highlights the need for further investigation, including future simulations of the 15 pre-AD aortic geometries using the fluid–structure interaction approach.

## 4. Results

A total of 215 patients with a type A acute aortic syndrome were identified from the local database. A total of 15 patients were included in the study with a confirmed type A aortic dissection and previous CT imaging for comparison analysis. There were 13 male patients (86.7%) and 2 female patients (13.3%). Of them, 11 patients (73.3%) died, with the most common cause of death being aortic dissection (9 patients). Baseline demographics are shown in Table 2.

The selected data of 15 patients were investigated based on the aortic diameter growth rate per year using the following formula:

$$Aortic\;diameter\;growth\;rate\;per\;year=\;\frac{Diameter\;post\;AD-Diameter\;pre\;AD}{CT\;scan\;duration\;post\;AD-CT\;scan\;duration\;pre\;AD}$$

The data indicate that some patients (e.g., P6 in group 1 and P3 in group 2) exhibited negative growth, while others showed almost no growth, such as P3 in group 1 and P5 in group 2. Table 3 presents the specific patient data, including final treatment and pre-AD imaging findings. The group classification was based on pre-AD imaging, with nine patients in group 1 categorized as normal and six patients in group 2 presenting with abnormal preconditions. These abnormal conditions were as follows: P1, P2, and P5 were diagnosed with dilated aortic root (DAR); P3 and P4 with dilated ascending aorta (DAA); and P6 with dilated aortic root with pseudoaneurysm (DARP). Clinically, a normal aortic wall was defined by a smooth, uniform, non-dilated structure on CT, with specific, age- and sex-dependent diameter limits (ascending, <40 mm; arch, <35 mm). The aortic size index (ASI), a calculation of aortic diameter divided by body surface area, offers a more personalized assessment of normality than absolute diameter. Generally, the ascending aorta is considered normal at <2.1 cm/m^2^. On the other hand, an abnormal wall is characterized by dilation (>1.5 times the expected normal diameter), increased wall thickness (>4 mm, often indicative of pathology), increased stiffness, or structural damage like dissection, aneurysms, or atherosclerotic plaque [6].

### 4.1. Pressure

**Figure 7:**
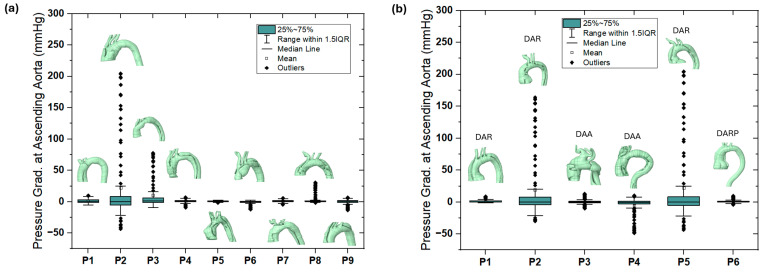
The pressure gradient (P-post-T–P-pre for the pressure waveform) at the ascending aorta for (**a**) the nine-patients with normal pre-condition (group 1) and (**b**) six-patients with abnormal pre-condition (group 2).

In this study, the pressure waveform at the ascending aorta was obtained from the CFD results and compared between the pre-AD and post-AD true lumen, alongside the maximum aortic diameter growth rate per year for both groups 1 and 2. Figure 7 shows the pressure gradient (based on the pressure waveform) at the ascending aorta for these patients following the equation (P-post-T–P-pre for the pressure waveform), measured in mmHg. The results indicate elevated pressure gradients for P2 in group 1, with the normal image findings, and P2 and P5 in group 2, with the DAR diagnosis. As shown in Figure 7a, patients P2 and P3 exhibited markedly elevated pressure gradients with wide variability and numerous outliers, suggesting abnormal hemodynamic loading conditions in the ascending aorta. In contrast, patients P4–P9 showed minimal or near-zero pressure gradients with narrow distributions, indicating more stable flow patterns. The associated 3D geometries illustrate how anatomical features, such as dilation, curvature, or branching patterns, influence these outcomes. Specifically, the complex geometries of P2 and P3 correspond to abnormal pressure elevations, whereas the smoother geometries of P4–P9 are associated with stable gradients. Figure 7b demonstrates that dilated aortic root (DAR) cases (especially P2 and P5) are associated with abnormally high and fluctuating pressure gradients, whereas cases of dilated ascending aorta (DAA) and pseudoaneurysm (DARP) show different patterns. These findings underscore that not all anatomical abnormalities produce the same hemodynamic consequences, and patient-specific geometry plays a crucial role in pressure distribution.

### 4.2. Wall Shear Stress Parameters

**Figure 8:**
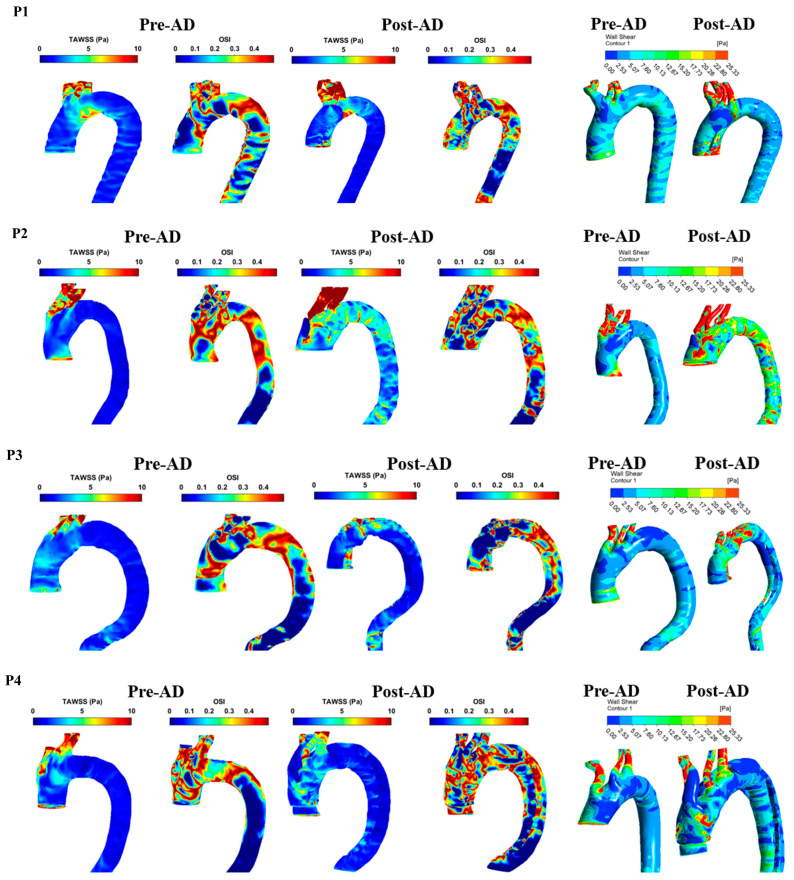

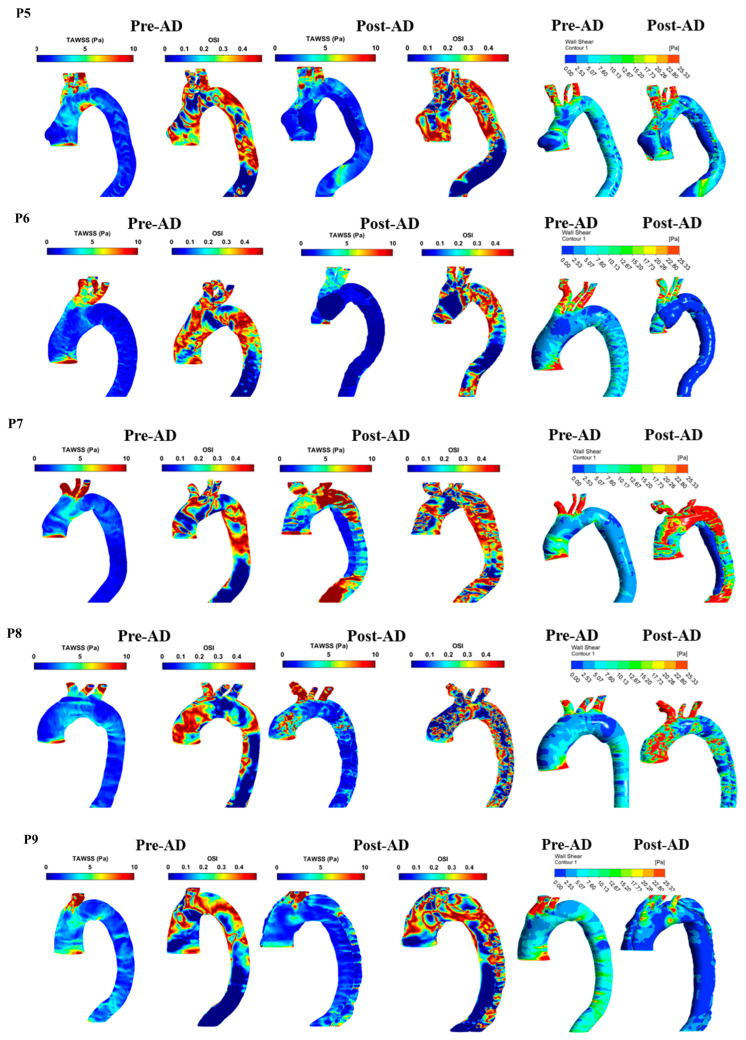
The TAWSS, OSI, and WSS for pre- and post-AD for P1–P9 (group 1).

The analysis of patient-specific hemodynamics revealed distinct variations in wall shear stress indices between pre-AD and post-AD aortic models. For WSS distribution, patients with ascending aortic involvement (P2, P3, and P6) displayed localized high-WSS zones that overlapped with eventual tear initiation sites. Post-AD, high WSS persisted around entry tears, while low and oscillatory WSS dominated within false lumens, as shown in Figure 8. Patients P5 and P7 showed more moderate and diffuse WSS distributions, whereas P4, P8, and P9 exhibited patchy high-WSS regions along the arch. Based on Manchester et al. [29], the total spatially averaged TAWSS was reported as 7.0 Pa in the ascending aorta and 3.8 Pa across the entire aorta. Petuchova and Maknickas [32] further identified that the normal physiological range of TAWSS in the ascending aorta of healthy individuals is typically 1.5–3.3 Pa during the cardiac cycle (mean 1.5 ± 0.3 Pa), while the OSI generally ranges between 0.15 and 0.3, with a mean value of 0.325 ± 0.025 [33,34,35,36]. In comparison, Table 4 shows that patients P2–P5 maintained TAWSS within the physiological range (2–4 Pa) and moderate OSI values (0.25–0.29), consistent with stable hemodynamics. In contrast, patients P1 and P6–P9 demonstrated pathological shear environments: P6 exhibited low TAWSS with elevated OSI, P7 presented extremely high TAWSS due to jet inflow, P8 showed abnormally low TAWSS reflecting stagnation, and P9 had reduced shear combined with increased oscillations. These results indicate that, despite normal pre-AD imaging, patients P6–P9 were exposed to abnormal shear conditions that may predispose them to false lumen propagation, thrombus formation, or further aortic remodeling.

**Figure 9:**
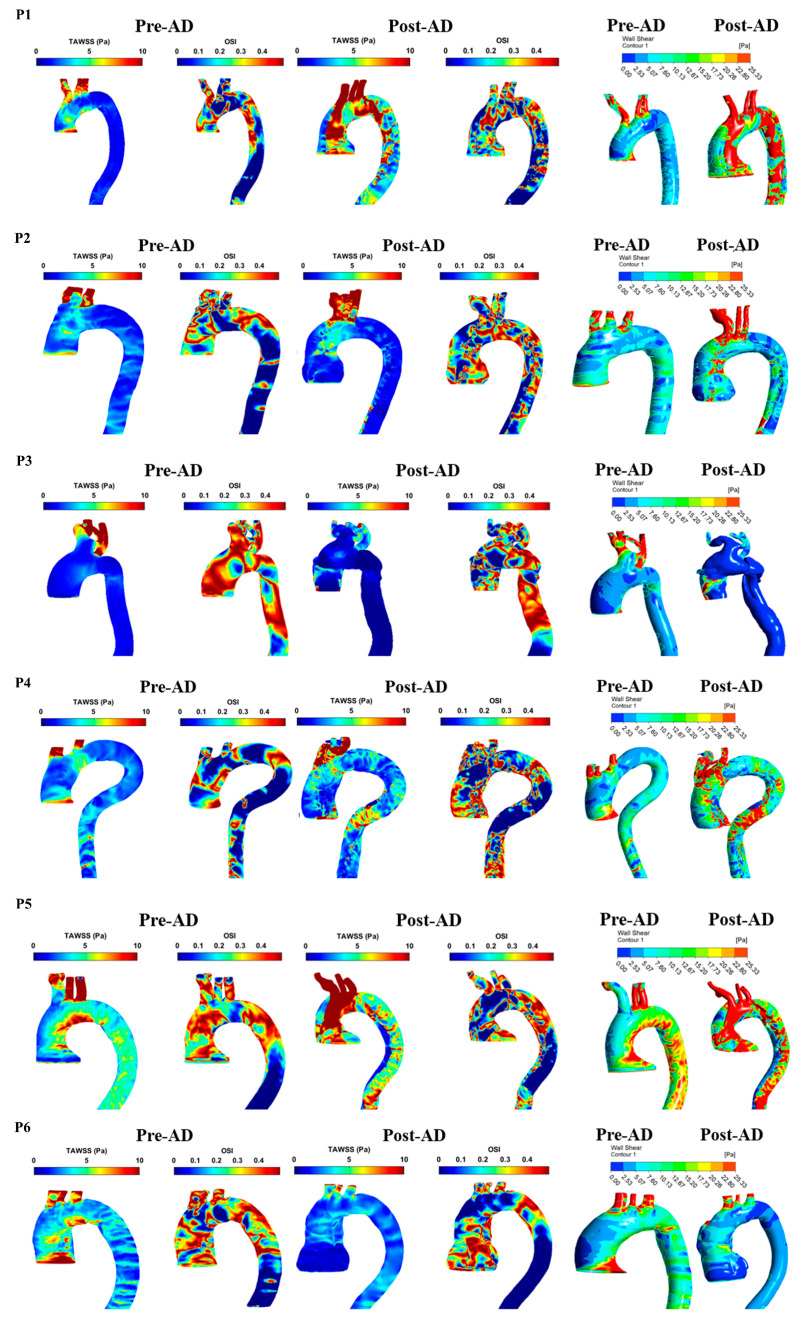
TAWSS, OSI, and WSS for pre- and post-AD for P1–P6 (group 2).

Figure 9 presents the WSS, TAWSS, and OSI distributions for group 2 in both pre-AD and post-AD states. This group, characterized by abnormal preconditions, showed clear WSS disturbances. DAR cases (P1, P2, and P5) exhibited elevated ascending aortic WSS, with P2 reaching critical levels due to jet inflow. DAA patients (P3 and P4) displayed patchy high-WSS regions that became more diffuse post-dissection. P6 (DARP) showed elevated WSS around the pseudoaneurysm. Overall, group 2 demonstrated more severe and localized WSS abnormalities, strongly associated with structural weakness and surgical intervention. Table 5 summarizes the TAWSS and OSI data from Figure 9. Compared to the physiological range of TAWSS (1.5–3.3 Pa) and OSI (0.15–0.3), most patients exceeded normal thresholds, indicating abnormal hemodynamics [32]. Post-AD TAWSS was markedly elevated in P1 and P2 (8.32 and 13.29 Pa), with P3 reaching critical levels (218.92 Pa). In contrast, P6 remained closer to normal values but showed signs of thrombus-prone or stagnant flow. OSI values generally fell within the expected range, though some patients (e.g., P1 and P4) displayed post-AD increases, suggesting heightened oscillatory shear.

**Figure 10:**
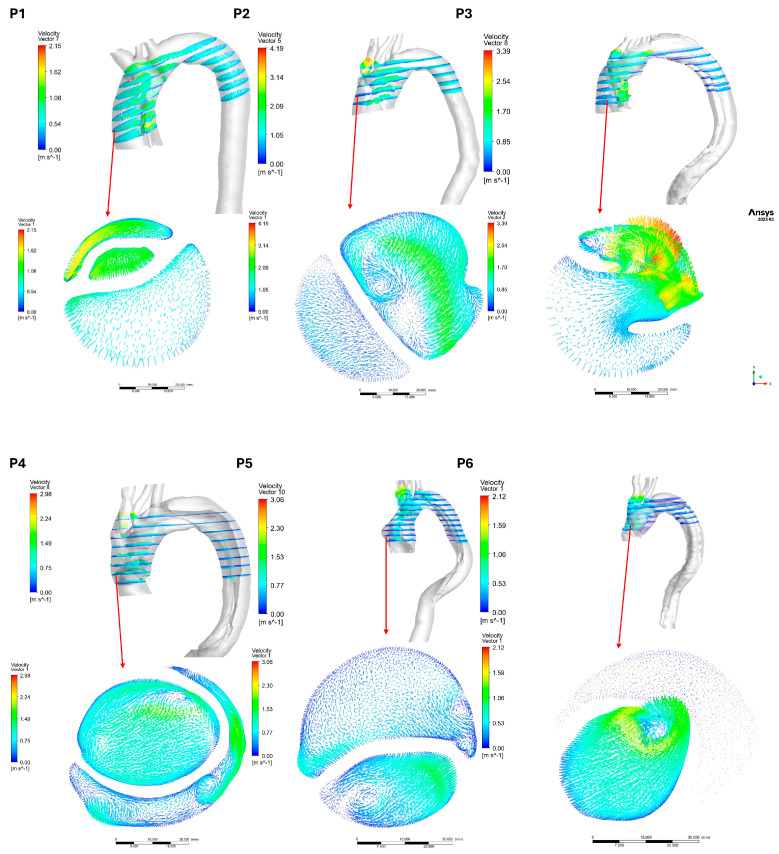

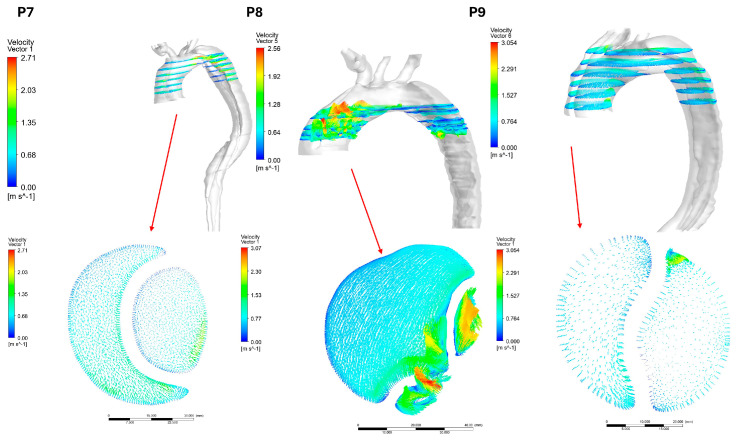
Post-AD velocity vectors for the nine patients in group 1 at the ascending aorta.

**Figure 11:**
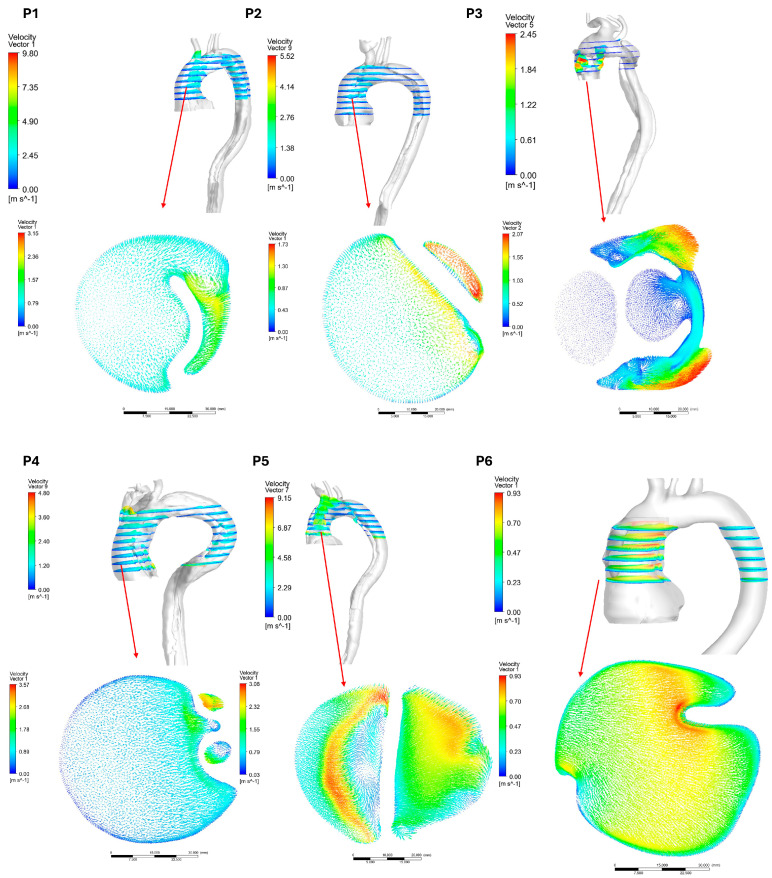
Post-AD velocity vectors for the six patients in group 2 at the ascending aorta.

The velocity vectors for the post-AD aortas illustrate the locations of blood flow separation, which give rise to both TL and FL. Figure 10 and Figure 11 show the velocity vectors on a plane at the ascending aorta for both groups in the post-AD state, with the marked points indicating the locations of the ascending pressure data presented in Figure 8. The results indicate that patients with advanced dissection development exhibited pronounced flow disturbances. CFD simulations of TAWSS and OSI contours revealed energy dissipation driven by blood flow recirculation, which is a major contributor to the increased risk of thrombus formation.

## 5. Discussion

This study investigates the role of WSS-derived parameters, such as TAWSS and OSI, in predicting the behavior of aortic dissection (AD) development based on data of 15 patients. The contours for these parameters were investigated with consideration of the aortic diameter growth rate per year. The results indicate a strong negative correlation: high pre-AD WSS resulted in lower diastolic pressure post-AD and low TAWSS, with a slight increase in OSI average values from 0.26 to 0.27 above the typical normal range of 0.15–0.3 (mean 0.325 ± 0.025) [32,33]. However, patients diagnosed with DAA show high TAWSS, reflecting increased shear forces exerted by blood flow on the aortic wall endothelium, leading to mechanical fatigue and weakening of the arterial wall, potentially resulting in aneurysms or dissections in the ascending aorta. High TAWSS contours at the TL increase the shear stress gradient at the dissection tear site. Additionally, OSI contours highlight directional changes in wall shear stress during the cardiac cycle, with an increase in its average values post-AD, adversely impacting the aortic wall, which predisposes it to thrombus formation and dysfunction.

In the analysis of 15 type A patients (nine with normal diagnoses and six with abnormal diagnoses), two distinct hemodynamic patterns were observed. In group 1 (normal diagnosis), pre-AD TAWSS values generally ranged between 2 and 4 Pa, slightly above the physiological range (1.5–3.3 Pa). Following dissection, TAWSS values diverged: some cases dropped below 1.5 Pa, indicative of stagnant, thrombus-prone flow, while others increased to approximately 9 Pa, consistent with jet-driven propagation into the false lumen. OSI values remained elevated (0.24–0.32), suggesting globally disturbed flow and potential for adverse remodeling. In contrast, group 2 (abnormal diagnosis) displayed more pronounced hemodynamic alterations. Several patients demonstrated critically high post-AD TAWSS values (10–219 Pa) at entry sites and aortic arch regions, while others dropped to <3 Pa, indicating stagnation and thrombus risk. OSI values (0.1–0.31) again reflected disturbed and oscillatory flow, with unstable patterns particularly evident in cases of elevated shear.

Our findings, consistent with prior literature, suggest that an annual aortic growth rate > 1.5 mm/year, combined with either entry-site TAWSS > 4–5 Pa or regional OSI ≥0.30, provides a practical threshold for risk stratification [9,20,23,25,26,29,34,35,36,37]. This framework distinguishes jet-driven rupture risk from stagnation-driven thrombus-prone remodeling. Accordingly, the results in Table 4 and Table 5, when compared with the annual diameter growth rate, highlight the potential of pre-TAWSS and maximum OSI values as early indicators of high-risk pre-AD development, particularly when the maximum aortic diameter growth exceeds 1.46 mm/year. Post-AD results further show a clear trend of OSI increasing in parallel with aortic diameter growth. Takeda et al. [23] reported that in eight patients with aortic dissection, type A cases exceeded 30 Pa WSS, corresponding to TAWSS ~3–5 Pa, with a maximum of 88.16 Pa (~5–9 Pa). Al-Rawi et al. [26] investigated one patient, showing pre-AD TAWSS ~5 Pa and post-AD ~10 Pa. Chi et al. [20] analyzed five type A patients, finding control WSS 5.6–6.5 Pa (TAWSS ~0.3–0.6 Pa), while patients reached up to 18.5 Pa near branch ostia. Osswald et al. [25] observed peak WSS up to 31.3 Pa (TAWSS ~1.5–3 Pa). Wang et al. [28] studied 15 type A cases with WSS > 20–40 Pa (TAWSS ~2–4 Pa). Zhu et al. [9] studied 17 post-repair patients and found that stable cases had TAWSS of 7–19 Pa, whereas unstable cases reached 16–33 Pa. Girardin et al. [34] reported peak systolic WSS of 25–40 Pa (TAWSS ~2–5 Pa) and reduced post-repair to ~1–3 Pa in 10 acute type A patients, consistent with [9,23]. Torii et al. [35] presented a single BAV aneurysm patient with WSS 18.6 Pa and TAWSS 5.3 Pa in the ascending aorta. Table 6 summarizes WSS and TAWSS findings across these studies.

**Figure 12:**
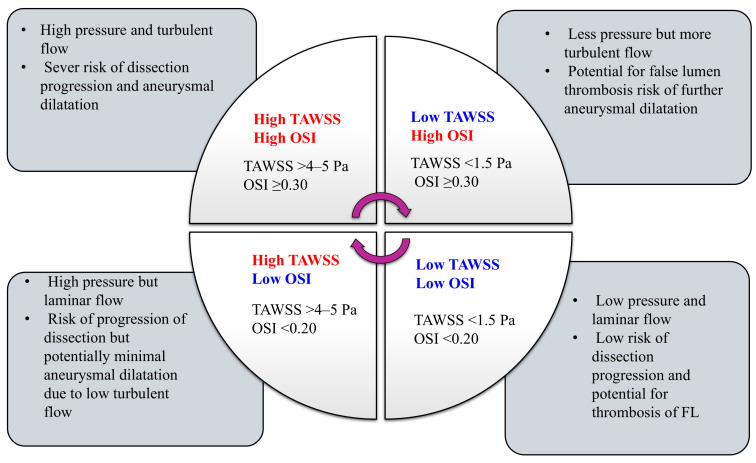
Clinical utility of CFD *hemodynamic* parameters of TAWSS and OSI.

The relationship between TAWSS and OSI can be conceptualized in four hemodynamic states, as illustrated in Figure 12. High TAWSS with high OSI reflects conditions of high pressure and turbulence, carrying a severe risk of dissection progression and aneurysmal dilatation. High TAWSS with low OSI indicates high but laminar shear forces, still carrying a risk of dissection propagation, although with potentially less aneurysmal dilatation due to minimal turbulence. Conversely, low TAWSS with high OSI suggests lower pressure but greater oscillatory flow, a pattern associated with false lumen thrombosis yet ongoing aneurysmal growth. Finally, low TAWSS with low OSI represents low-pressure, laminar environments, corresponding to a lower risk of dissection progression and favoring false lumen thrombosis and stabilization. This quadrant framework provides a practical approach for interpreting CFD-derived hemodynamic metrics and stratifying patients according to progression versus stabilization risk. For example, in patient 1 (group 1), a 1.27 mm/year growth rate with a normal baseline CT might have previously warranted observational follow-up. Using our proposed threshold (TAWSS > 4–5 Pa and OSI ≥ 0.3), this patient would have received earlier surgical intervention, mitigating the risk of rapid progression to aortic dissection.

Clinical assessment is transitioning from traditional, size-centric definitions toward a more sophisticated model that accounts for hemodynamic wall stress, which this study aims to establish. Further, a clear comparison between traditional diameter-based thresholds and our proposed CFD threshold is necessary to demonstrate the potential added value of hemodynamic assessment. However, we would like to highlight the challenges of a direct comparison based on several factors: Firstly, our results reinforce that not all anatomical abnormalities produce the same consequences. Secondly, although our study offers a valuable analysis of 15 patients, the small sample size means our findings should be interpreted as a pilot validation. Finally, our current CFD simulations assume rigid walls, neglecting aortic wall–blood interaction, which means that incorporating fluid–structure interaction (FSI) would likely reduce the overestimation of wall shear stress. Future work will employ a larger cohort to validate our findings across diverse patient-specific geometries, utilizing advanced fluid–structure interaction (FSI) modeling.

The preliminary results demonstrate promising outcomes with a low computational cost. However, several limitations exist in this study, which will be addressed in future work, including the rigid-wall assumption and the use of literature-based boundary conditions. Future studies will incorporate a three-element Windkessel model combined with two-way fluid–structure interaction (FSI) for these 15 patients [36,37].

The retrospective design may introduce selection bias, as the CT scan data were collected for clinical purposes rather than standardized CFD research protocols, which limited the number of available cases for this study. Therefore, future work will include prospective data collection and standardized imaging specifically designed for CFD analysis, incorporating patient-specific boundary conditions.

## 6. Conclusions

Overall, our results reinforce the dual role of hemodynamics in aortic dissection: focal jet-driven TAWSS elevations increase wall stress at entry sites, while low TAWSS/high OSI environments within the false lumen promote thrombus formation and adverse remodeling. Linking these indices to simple clinical markers, such as an aortic growth rate exceeding 1.5 mm/year, provides a promising framework for early risk stratification to support both conservative monitoring and surgical decision-making. Identifying TAWSS and OSI thresholds can support surgical decision-making in type A dissection. Patients with low TAWSS (<1.5–2 Pa), particularly in the false lumen, are at increased risk of stagnation and thrombus formation, suggesting they may be managed with ongoing surveillance. Conversely, patients with high TAWSS (>5–10 Pa), especially when combined with disturbed flow (OSI > 0.3), face a higher risk of false lumen propagation and rupture, justifying earlier or prophylactic surgical intervention to prevent adverse outcomes. While promising, these results are preliminary; the mentioned limitations hinder firm conclusions regarding clinical utility. These thresholds require validation in larger studies before they can inform clinical decision-making. Future work will incorporate a one-way fluid–structure interaction (FSI) method between the arterial wall and blood flow, together with patient-specific boundary conditions (blood flow waveforms and pressure readings) modeled using the three-element Windkessel approach.

## Figures and Tables

**Table 1:** The four meshes for patient 7 (group 2) show the maximum pressure (at the ascending aorta), the maximum pressure (at the descending aorta), the minimum orthogonal quality, the maximum aspect ratio, and the wall $Y^{+}$.

#	Total Number of Cells	P@Ascending (mmHg)	%	P@Descending (mmHg)	%	Min Orthogonal Quality	Max Aspect Ratio	Wall Y^+^
M1	4,599,484	180.25	0.41%	176.8	0.29%	0.2355	13.49	0.88
M2	963,421	181	0.10%	177.32	0.05%	0.2459	13.1	1.5
M3	569,096	181.19	10.95%	177.4	11.30%	0.2382	14	1.9
M4	30,021	203.47		200.01		0.1005	64.44	5.1

**Table 2:** Baseline demographics of the included patients.

Data	Count (%)
Male	12 (86.7)
Female	2 (13.3)
Ethnicity New Zealand European New Zealand Māori	8 (53.3) 7 (46.7)
Ischaemic heart disease	5 (33.3)
Hypertension	11 (73.3)
Atrial fibrillation	3 (20.0)
Previous stroke/transient ischaemic attack (TIA)	4 (26.7)
Chronic obstructive pulmonary disease (COPD)	4 (26.7)
Diabetes mellitus	3 (20.0)
Congestive heart failure	5 (33.3)
Presence of a bicuspid aortic valve	0 (0.0)
Bovine arch	2 (13.3)
Vertebral artery arch origin	2 (13.3)
Familial aortopathy	1 (6.7)
Known connective tissue disease	0 (0.0)
Use of cocaine/methamphetamines	1 (6.7)
Previous cardiac surgery	5 (33.3)
Previous cardiac catheterization	4 (26.7)
Smoking Current Ex No	3 (20.0) 7 (46.7) 5 (33.3)
Previous aortic dissection	1 (6.7)

**Table 3:** The data of 15 patients pre- and post-AD type A, including medical diagnoses based on the CT scan images (nine patients with normal preconditions in group 1 and six patients with abnormal preconditions in group 2).

Patient	Gender	Age at First Diagnosis	Imaging Finding Ascending Aorta	Pre-AD Aortic Diameter (mm)	Post-AD Aortic Diameter (mm)	Growth Rate (mm/Year)	Final Treatment Received
Group 1 with normal precondition							
P1	M	60	Normal	37	48	1.27	Surgery
P2	F	70	Normal	39	53	2.31	Conservative
P3	M	54	Normal	42	42	0	Conservative
P4	M	56	Normal	43	51	6.27	Conservative
P5	M	76	Normal	44	57	2.65	Conservative
P6	M	73	Normal	46	40	−1.24	Conservative
P7	M	63	Normal	46	51	1.46	Conservative
P8	M	32	Normal	48	57	1.24	Conservative
P9	F	69	Normal	48	49	0.29	Conservative
Group 2 with abnormal precondition							
P1	M	46	DAR	51	62	1.9	Surgery
P2	M	46	DAR	54	64	31.86	Surgery
P3	M	62	DAA	55	45	−1.62	Surgery
P4	M	86	DAA	58	60	144	Conservative
P5	M	32	DAR	66	66	0	Surgery
P6	M	66	DARP	69	81	17.63	Conservative

**Table 4:** TAWSS and OSI pre- and post-AD type A for the nine patients (group 1) with normal pre-AD diagnoses.

#	Pre-TAWSS (Pa)	Post-TAWSS (Pa)	Comments on the Pre	OSI-Pre (-)	OSI-Post (-)	Comments
1	4.79	4.49	Uniform distribution of shear forces along the aorta, indicating weakened inflow into the FL.	0.2546	0.2705	Stagnation in the FL. High shear forces at the outflow increase thrombus risk.
2	2.85	4.88	Uniform distribution. Strong jet inflow.	0.2644	0.2591	Active flow propagation through the FL. Some flow dissipation causes turbulent flow.
3	3.72	3.22	Uniform distribution. Active flow propagation.	0.2496	0.2598	Dissipation at the outlet. Some flow dissipation causes turbulent flow.
4	2.38	2.07	Uniform distribution, reflecting inflow into the FL.	0.2577	0.27	Partial dissipation. Low-energy outflow.
5	2.59	2.18	Uniform flow. Weak inflow into the FL.	0.2828	0.2971	Low shear stresses, but still stagnant flow.
6	2.51	1.2	Exceeded the threshold; below normal range (mean 1.5 ± 0.3).	0.2471	0.3201	Below normal range (mean 0.325 ± 0.025).
7	2.44	9.33	Exceeded the threshold. Strong jet inflow.	0.2558	0.2765	Active flow propagation through the FL.
8	2.61	0.16	Exceeded the threshold. Active flow propagation.	0.253	0.2364	Dissipation at the outlet.
9	4.41	3.56	Exceeded the threshold. Weak inflow into the FL.	0.244	0.2647	Low shear stresses.

**Table 5:** TAWSS and OSI pre- and post-AD type A for the six patients (group 2) with abnormal diagnoses.

#	Pre-TAWSS (Pa)	Post-TAWSS (Pa)	Comments	OSI-Pre (-)	OSI-Post (-)	Comments
1	5.35	8.32	Moderate shear stresses with very low oscillation.	0.2495	0.3102	Increase in shear stress but further reduction in its oscillation.
2	2.77	13.29	Normal shear stress with slightly elevated oscillation.	0.1499	0.1057	Stresses increased with the reduction in oscillation due to thrombus formation from the pre-AD state.
3	37.93	218.92	High shear stresses with moderate oscillation.	0.079	0.2349	Critical condition due to highly elevated shear tresses with high oscillation.
4	3.23	4.38	Balanced shear stresses on the artery wall with oscillation.	0.2554	0.2819	Increase in stresses and oscillation; post-AD flow active at the ascending aorta.
5	5.17	10.38	Elevated shear stresses and clear oscillatory behavior.	0.272–0.4	0.1–0.2702	Increased stresses at the locations of the ascending aorta and aortic arch.
6	3.67	2.35	Still above the threshold of stresses. The potential of a thrombus-prone region is evident at the ascending aorta.	0.25299	0.2617	Drop in shear stresses and oscillation.

**Table 6:** The literature data for WSS and TAWSS metric.

Study	Cohort	Reported Metric(s)	Peak WSS (Pa)	TAWSS (Pa) (Reported or Estimated)	Notes
Takeda et al., 2022 [23]	11 (3 healthy, 7 type A, 1 type B)	WSS	Type A: 30–88.16	~3–9 (est.)	5/7 type A > 38 Pa (damage threshold); highest peak 88 Pa
Chi et al., 2017 [20]	5 premorbid type A, 2 controls	WSS	9.18–18.50	~0.5–2 (est.)	Pre-AD ascending aortas; lower stresses than Takeda
Al-Rawi et al., 2024 [26]	Single CFD case (pre-/post-type A)	TAWSS (direct)	–	Pre-AD: up to 5; post-AD: up to 10	Directly reported TAWSS; highest in arch and supra-aortic branches
Osswald et al., 2017 [18]	20 (10 RTAD, 10 control)	WSS	RTAD: 5.15–31.30; Controls: 3.82–12.07	RTAD: 0.8–3 (est.); controls: 0.3–1.2 (est.)	High WSS near the subclavian in RTAD patients; predictor of retrograde type A
Wang, T. et al., 2021 [28]	15 (rupture vs. unruptured type A vs. healthy)	Swirling strength, pressure	Not reported	Ruptured ~2–4 (est.); unruptured ~1–3 (est.); healthy ~1.5–3.3 (lit.)	Used swirling strength as a surrogate; rupture cases had the highest wall stress
Zhu, Y. et al., 2021 [9]	17 (9 stable, 8 unstable post-type A repair)	TAWSS (direct)	–	Stable: 7–19; unstable: 16.5–32.8	High TAWSS at the primary tear edge predicts dilatation risk

## Data Availability

Due to patient privacy, the datasets generated and analyzed during the study are not publicly available.

## Abbreviations

The following abbreviations are used in this manuscript:

AD	Aortic Dissection
CFD	Computational Fluid Dynamics
CT	Computed Tomography
DAA	Dilated Ascending Aorta
DAR	Dilated Aortic Root
DARP	Dilated Aortic Root with Pseudoaneurysm
IMH	Intramural Hematoma
OSI	Oscillatory Shear Index
PAU	Penetrating Aortic Ulcer
RANS	Reynolds-Averaged Navier–Stokes
SST	Shear Stress Transport
STL	Standard Tessellation Language
TAAD	Type A Aortic Dissection
TAWSS	Time-Average Wall Shear Stress
WSS	Wall Shear Stress

## References

[1] Haran C., Ghafouri K., Xu W., Hayes I., Stiles M., Khashram M. (2023). Prevalence of genetically triggered aortopathy in acute aortic syndrome in Aotearoa New Zealand. Eur. J. Vasc. Endovasc. Surg..

[2] Rolf-Pissarczyk M., Schussnig R., Fries T., Fleischmann D., Elefteriades J.A., Humphrey J.D., Holzapfel G.A. (2024). Mechanisms of aortic dissection: From pathological changes to experimental and in silico models. Prog. Mater. Sci..

[3] Xu W., Mani K., Khashram M. (2021). Ethnic differences in incidence and outcomes of acute aortic syndromes in the Midland region of New Zealand. J. Vasc. Surg..

[4] Aranda-Michel E., Bianco V., Yousef S., Brown J., Dai Y., Serna-Gallegos D., Hoskoppal A., Sultan I. (2022). National trends in thoracic aortic aneurysms and dissections in patients with Marfans and Ehlers Danlos syndrome. J. Card. Surg..

[5] Xu W., Haran C., Dean A., Lim E., Bernau O., Mani K., Khanafer A., Pitama S., Khashram M. (2023). Acute aortic syndrome: Nationwide study of epidemiology, management, and outcomes. Br. J. Surg..

[6] Isselbacher E.M., Preventza O., Black J.H., Augoustides J.G., Beck A.W., Bolen M.A., Braverman A.C., Bray B.E., Brown Z., Chen E.P. (2022). 2022 ACC/AHA Guideline for the diagnosis and management of aortic disease: A report of the American Heart Association/American College of Cardiology Joint Committee on Clinical Practice Guidelines. J. Am. Coll. Cardiol..

[7] Wen J., Huang Q., Chen X., Zhang K., Peng L. (2025). Impact of Aortic Branch Retention Strategies on Thrombus Growth Prediction in Type B Aortic Dissection: A Hemodynamic Study. Comput. Methods Programs Biomed..

[8] Poullis M.P., Warwick R., Oo A., Poole R.J. (2008). Ascending aortic curvature as an independent risk factor for type A dissection, and ascending aortic aneurysm formation: A mathematical model. Eur. J. Cardio-Thorac. Surg..

[9] Zhu Y., Xu X.Y., Rosendahl U., Pepper J., Mirsadraee S. (2022). Advanced risk prediction for aortic dissection patients using imaging-based computational flow analysis. Clin. Radiol..

[10] Wei Y., Li D., Weng C., Wang J., Yuan D., Zheng T. (2024). Hypertension-Induced Biomechanical Modifications in the Aortic Wall and Their Role in Stanford Type B Aortic Dissection. Biomedicines.

[11] Lee G., Heo W., Lee Y., Kim T., Huh H., Song S., Ha H. (2023). Fluid–structure interaction simulation of visceral perfusion and impact of different cannulation methods on aortic dissection. Sci. Rep..

[12] Gramigna V., Palumbo A., Rossi M., Fragomeni G. (2023). A computational fluid dynamics study to compare two types of arterial cannulae for cardiopulmonary bypass. Fluids.

[13] Alimohammadi M., Sherwood J.M., Karimpour M., Agu O., Balabani S., Díaz-Zuccarini V. (2015). Aortic dissection simulation models for clinical support: Fluid-structure interaction vs. rigid wall models. BioMed. Eng. OnLine.

[14] Tsai T., Guo X., Kageyama S., Lim R.P., Tanaka K., De Mey J., La Meir M., Onuma Y., Poon E.K., Serruys P.W. (2024). Managing Iatrogenic Aortic Dissection: Insight from 3D-holographic Imaging and CT Computational Fluid Dynamic Simulations. J. Am. Coll. Cardiol..

[15] Wang Z., Tian S., Yu L., Ma X., Xing Y., Ma X. (2021). The role of the aortic area in type A aortic dissection. Biomed. Signal Process. Control.

[16] Karmonik C., Bismuth J., Shah D., Davies M., Purdy D., Lumsden A. (2011). Computational study of haemodynamic effects of Entry- and Exit-Tear coverage in a DEBAKEY Type III aortic dissection: Technical report. Eur. J. Vasc. Endovasc. Surg..

[17] Harris C., Croce B., Cao C. (2016). Type A aortic dissection. Ann. Cardiothorac. Surg..

[18] Osswald A., Karmonik C., Anderson J., Rengier F., Karck M., Engelke J., Kallenbach K., Kotelis D., Partovi S., Böckler D. (2017). Elevated wall shear stress in aortic type B dissection may relate to retrograde aortic Type A dissection: A Computational Fluid dynamics pilot study. Eur. J. Vasc. Endovasc. Surg..

[19] Shi Y., Zhu M., Chang Y., Qiao H., Liu Y. (2016). The risk of stanford type-A aortic dissection with different tear size and location: A numerical study. BioMed. Eng. OnLine.

[20] Chi Q., He Y., Luan Y., Qin K., Mu L. (2017). Numerical analysis of wall shear stress in ascending aorta before tearing in type A aortic dissection. Comput. Biol. Med..

[21] Shad R., Kong S., Fong R., Quach N., Kasinpila P., Bowles C., Lee A., Hiesinger W. (2021). Computational fluid dynamics simulations to predict false lumen enlargement after surgical repair of Type-A aortic dissection. Semin. Thorac. Cardiovasc. Surg..

[22] Wen J., Huang H., Su Z., Jiang L., Gao Q., Chen X., Yan T., Peng L. (2022). Predicting the Risk of Type B Aortic Dissection Using Hemodynamic Parameters in Aortic Arches: A Comparative Study between Healthy and Repaired Aortas. Comput. Methods Programs Biomed..

[23] Takeda R., Sato F., Yokoyama H., Sasaki K., Oshima N., Kuroda A., Takashima H., Li C., Honda S., Kamiya H. (2021). Investigations into the Potential of Using Open Source CFD to Analyze the Differences in Hemodynamic Parameters for Aortic Dissections (Healthy versus Stanford Type A and B). Ann. Vasc. Surg..

[24] Czerny M., Grabenwöger M., Berger T., Aboyans V., Della Corte A., Chen E.P., Desai N.D., Dumfarth J., Elefteriades J.A., Etz C.D. (2024). EACTS/STS guidelines for diagnosing and treating acute and chronic syndromes of the aortic organ. Eur. J. Cardio-Thorac. Surg..

[25] Slicer Community. 3D Slicer [Computer Software]. https://www.slicer.org.

[26] Al-Rawi M., Belkacemi D., Lim E.T.A., Khashram M. (2024). Investigation of type A aortic dissection using computational modelling. Biomedicines.

[27] Al-Jumaily A.M., Al-Rawi M., Belkacemi D., Sascău R.A., Stătescu C., Țurcanu F.-E., Anghel L. (2024). Computational Modeling Approach to Profile Hemodynamical Behavior in a Healthy Aorta. Bioengineering.

[28] Wang T., Quast C., Bönner F., Kelm M., Zeus T., Lemainque T., Steinseifer U., Neidlin M. (2025). Investigation of hemodynamic bulk flow patterns caused by aortic stenosis using a combined 4D Flow MRI-CFD framework. PLoS Computat. Biol..

[29] Manchester E.L., Pirola S., Salmasi M.Y., O’Regan D.P., Athanasiou T., Xu X.Y. (2021). Analysis of Turbulence Effects in a Patient-Specific Aorta with Aortic Valve Stenosis. Cardiovasc. Eng. Technol..

[30] Benim A., Nahavandi A., Assmann A., Schubert D., Feindt P., Suh S. (2010). Simulation of blood flow in human aorta with emphasis on outlet boundary conditions. Appl. Math. Model..

[31] Al-Rawi M., Al-Jumaily A.M., Belkacemi D. (2022). Non-invasive diagnostics of blockage growth in the descending aorta-computational approach. Med. Biol. Eng. Comput..

[32] Petuchova A., Maknickas A. (2022). Computational analysis of aortic haemodynamics in the presence of ascending aortic aneurysm. Technol. Health Care.

[33] Condemi F., Campisi S., Viallon M., Croisille P., Fuzelier J.F., Avril S. (2018). Ascending thoracic aorta aneurysm repair induces positive hemodynamic outcomes in a patient with unchanged bicuspid aortic valve. J. Biomech..

[34] Girardin L., Lind N., von Tengg-Kobligk H., Balabani S., Díaz-Zuccarini V. (2025). Impact of Residual Intimal Flap Displacement Post-TEVAR on TBAD Haemodynamics in Compliant, Patient-specific CFD Simulations Informed by MRI. Ann. Biomed. Eng..

[35] Torii R., Kalantzi M., Theodoropoulos S., Sarathchandra P., Xu X.Y., Yacoub M.H. (2013). Predicting Impending Rupture of the Ascending Aorta with Bicuspid Aortic Valve: Spatiotemporal Flow and Wall Shear Stress. JACC Cardiovasc. Imaging.

[36] Al-Rawi M., Lim E.T.A., Khashram M. Two-Way Fluid-Structure Interaction to Assess the Development of Aortic Dissection Type A. Proceedings of the 2025 International Mechanical Engineering Congress and Exposition.

[37] Al-Rawi M., Lim E.T.A., Khashram M. Boundary Conditions for Aorta Computational Modelling Using the Literature Data. Proceedings of the 2025 International Mechanical Engineering Congress and Exposition.

